# A Photoalkylative Fluorogenic Probe of Guttiferone A for Live Cell Imaging and Proteome Labeling in *Plasmodium falciparum*

**DOI:** 10.3390/molecules25215139

**Published:** 2020-11-04

**Authors:** Romain Duval, Kevin Cottet, Magali Blaud, Anaïs Merckx, Sandrine Houzé, Philippe Grellier, Marie-Christine Lallemand, Sylvie Michel

**Affiliations:** 1 Université de Paris, MERIT, IRD, F-75006 Paris, France; anais.merckx@parisdescartes.fr (A.M.); sandrine.houze@parisdescartes.fr (S.H.); 2 Université de Paris, CiTCoM, CNRS, F-75006 Paris, France; kevin.cottet@hotmail.fr (K.C.); magali.blaud@parisdescartes.fr (M.B.); marie-christine.lallemand@parisdescartes.fr (M.-C.L.); sylvie.michel@parisdescartes.fr (S.M.); 3 CNR du Paludisme, AP-HP, Hôpital Bichat—Claude-Bernard, F-75018 Paris, France; 4 Unité Molécules de Communication et Adaptation des Microorganismes (MCAM), UMR 7245, CNRS Muséum National d’Histoire Naturelle, F-75005 Paris, France; philippe.grellier@mnhn.fr

**Keywords:** Guttiferone A, *Plasmodium falciparum*, 7-azidocoumarin, photoactivation, fluorogenesis

## Abstract

Guttiferone A (GA) **1**, a polycyclic polyprenylated acylphloroglucinol (PPAP) isolated from the plant *Symphonia globulifera* (Clusiaceae), constitutes a novel hit in antimalarial drug discovery. PPAPs do not possess identified biochemical targets in malarial parasites up to now. Towards this aim, we designed and evaluated a natural product-derived photoactivatable probe AZC-GA **5**, embedding a photoalkylative fluorogenic motif of the 7-azidocoumarin (AZC) type, devoted to studying the affinity proteins interacting with GA in *Plasmodium falciparum*. Probe **5** manifested a number of positive functional and biological features, such as (i) inhibitory activity in vitro against *P. falciparum* blood-stages that was superimposable to that of GA **1**, dose–response photoalkylative fluorogenic properties (ii) in model conditions using bovine serum albumin (BSA) as an affinity protein surrogate, (iii) in live *P. falciparum*-infected erythrocytes, and (iv) in fresh *P. falciparum* cell lysate. Fluorogenic signals by photoactivated AZC-GA **5** in biological settings were markedly abolished in the presence of excess GA **1** as a competitor, indicating significant pharmacological specificity of the designed molecular probe relative to the native PPAP. These results open the way to identify the detected plasmodial proteins as putative drug targets for the natural product **1** by means of proteomic analysis.

## 1. Introduction

Malaria, a devastating tropical disease caused by protozoan parasites of the genus *Plasmodium*, is currently responsible for ca. 400,000 deaths annually [1]. Artemisinin derivatives still constitute the frontline antimalarials prescribed worldwide, and are estimated to have saved millions of lives during the last two decades due to their fast curative action, particularly against the most deadly and Africa-prevalent *P. falciparum* [2]. These progresses are threatened by the spread of artemisinin-resistant parasite strains, which emerged one decade ago in South-East Asia [3]. This phenomenon is a worrying continuum of the history of chemoresistance to most major antimalarials [4]. In this context, the search for original antiplasmodial compounds with novel mechanisms of action and biochemical targets is urgently needed. Natural compounds constitute an especially relevant source of antimalarial drugs due to the fact that these compounds rarely inhibit one given pathway but rather multiple ones within target cells [5,6], thus drastically limiting the emergence of resistant mutants, which would require concomitant mutations at the level of several genes [7,8]. 

**Figure 1:**
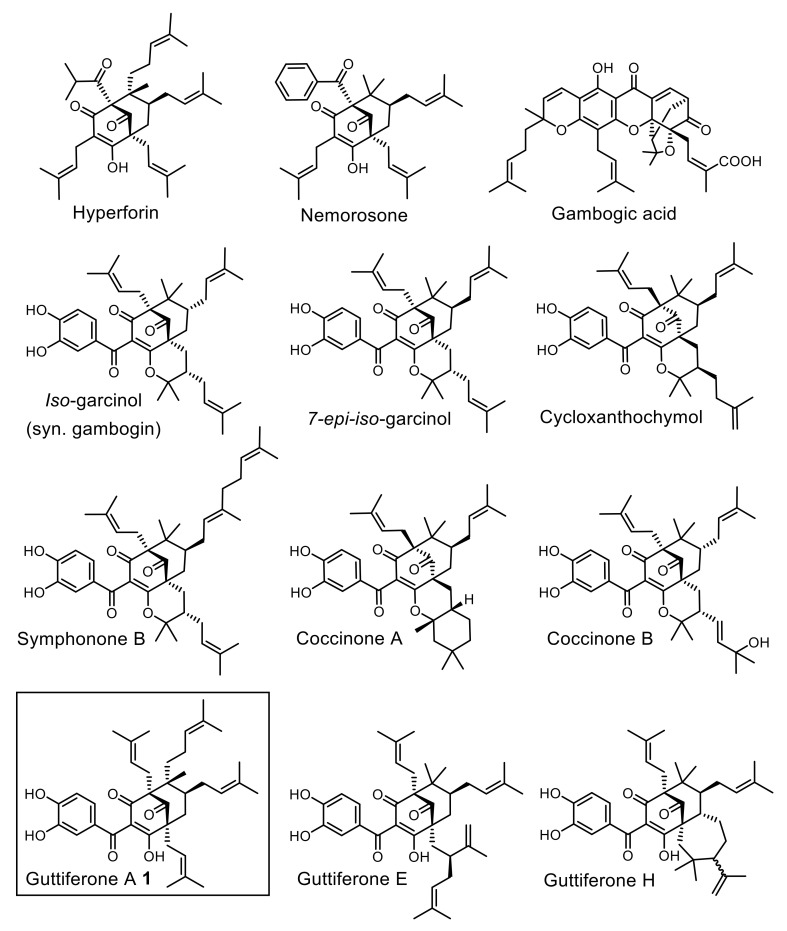
Representative antimalarial PPAPs (IC_50_ = 0.2 to ca. 5 µM in vitro).

Guttiferone A **1** (GA) is a polycyclic polyprenylated acylphloroglucinol (PPAP) isolated from *Symphonia globulifera* (Clusiaceae) [9,10]. **1** possesses a broad spectrum of medicinal properties in vitro, including significant inhibitory activity against several *P. falciparum* laboratory strains (IC_50_ = 0.5 to 3.3 µM) [11,12,13,14]. Antimalarial action is a common trend amongst various PPAPs (Figure 1) [13,14,15,16,17,18,19], suggesting that these natural products interact with promiscuous classes of targets within the parasite—possibly sirtuins [20,21,22] and mitochondrial proteins [19,23,24,25]—which, however, remained elusive up to now.

## 2. Results and Discussion

### 2.1. Probe Principle and Design 

**Figure 2:**
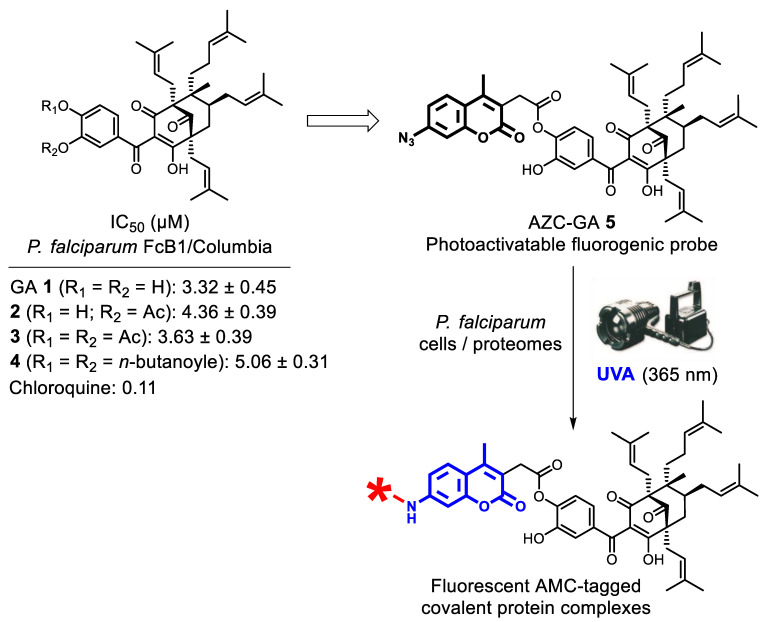
SARs in ester series [11] for the design of photoactivatable probe AZC-GA **5**, and its principle of fluorogenic covalent capture of affinity proteins (star = protein).

PPAPs correspond to unprecedented scaffolds in antiplasmodial pharmacochemistry, GA **1** itself featuring a compact hexa-oxygenated core decorated with four prenyle chains. No pharmacophore or parasite targets for this novel hit could yet be identified. This prompted us to design, synthesize, and evaluate a GA-derived probe to address these questions using microscopic imaging and chemical proteomic strategies. Regarding structure–activity relationships (SARs) in the series, a number of preliminary modifications performed on the catechol subunit of **1** previously showed that *mono*- or *bis*-esterification of the catechol even by some relatively bulky moieties were maintaining the antiplasmodial activity of the natural PPAP (Figure 2) [11]. This opened the way to esterification with large interactomic labels, such as fluorophores, and we chose to derivatize **1** with a 7-azidocoumarin (AZC) photoalkylative fluorogenic label, previously described per se as a photoaffinity inhibitor of a human phenol sulfotransferase [26]. The key property expected from the target probe AZC-GA **5** is the formation of fluorescent 7-aminocoumarin (AMC) covalent complexes with protein of high affinity upon UVA irradiation, which could be detected using live cell imaging and sodium dodecylesulfate-polyacrylamide gel electrophoresis (SDS-PAGE) [26,27] (Figure 2).

### 2.2. Probe Synthesis and In Vitro Activity against P. falciparum Blood-Stages

**Figure 3:**
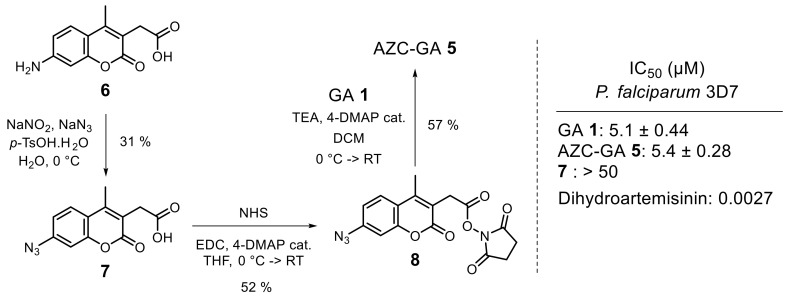
Synthesis and in vitro antiplasmodial activity of AZC-GA **2**.

Probe **5** was easily assembled by the regioselective *mono*-esterification of GA **1** with the activated ester AZC-NHS **8** in basic conditions, which proceeded as expected via the most nucleophilic *p*-phenolate of the PPAP with a 57% yield. AZC-NHS **8** was obtained from commercial 7-amino-4-methyl-3-coumarinylacetic acid **6** following a two-step sequence of Sandmeyer-type aromatic azidation and NHS esterification, with acceptable yields. The proper design of AZC-GA **5** as a chemical probe according to existing SARs was indicated by its in vitro IC_50_ value on the *P. falciparum* 3D7 strain, which was superimposable to that of native GA **1**, the AZC label **7**, on the other hand, being inactive in the range tested (Figure 3). 

### 2.3. Proof-of-Principle of the Photoactivated Fluorogenic Covalent Labeling with AZC-GA 5 on BSA 

**Figure 4:**
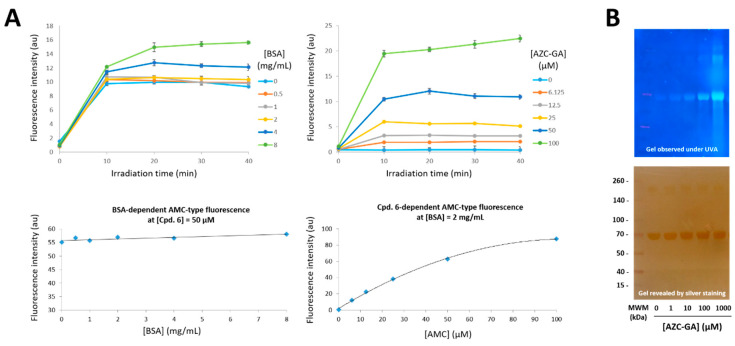
Fluorimetric and SDS-PAGE analysis of the fluorogenic photoalkylation of BSA by AZC-GA **5**. (**A**): 50 µM AZC-GA **5** in RIPA lysis buffer were used for the dose–response photoalkylation of BSA (0 to 8 mg/mL) at various irradiation times (left top panel). 2 mg/mL BSA in RIPA lysis buffer were used for the dose–response photoalkylation by AZC-GA **5** (0 to 100 µM) at various irradiation times (right top panel). Dose–response variation of AMC-type fluorescence was assessed using BSA (0 to 8 mg/mL, left bottom panel) and non-photoactivated **6** (0 to 100 µM (right bottom panel), taking **6** as exemplary of the AMC fluorophore present in AMC-GA-BSA photoadducts (Figure 2). Fluorimetric analysis was performed on a Xenius XML spectrofluorimeter (SAFAS, Monaco) at 23 °C using the following detection settings: λ_EX_ = 360 nm, λ_EM_ = 448 nm. Values represent the mean of three sequential measurements (interval time = 5 s). Standard deviations are systematically shown on the plots but are often invisible due to their close-to-zero values. (**B**): 2 mg/mL BSA in RIPA lysis buffer was used for the dose–response photoalkylation by AZC-GA **5** under UVA irradiation for 20 min. Noteworthy, BSA shows slight blue autofluorescence in the absence of AZC-GA **5** (lane 0 µM). Fluorescence revelation was performed using the UV bed of an E-Box VX2 scanner (Vilber-Lourmat, Collegien, France) and pictured as seen with the naked eye. All photoactivations were performed on ice (0–4 °C). MWM = molecular weight marker.

As a first step towards the target study, validation of the photoactivation principle and definining of its conditions and kinetics for AZC-GA **5** was performed using bovine serum albumin (BSA) as an affinity protein surrogate. Blood albumins are major small-molecule plasmatic carriers in animals [28,29]. They possess non-specific affinity towards a broad range of drugs and natural products [30], making them ideal choices as surrogates for target proteins in model studies [31]. By using reciprocal gradients of AZC-GA **5** and BSA, we demonstrated that **5**, a profluorescent non-emitting species, was forming stable AMC-BSA fluorescent complexes that could be detected by fluorimetric and SDS-PAGE experiments (Figure 4). Regarding fluorimetric analysis, maximum fluorescence intensity was obtained as expected in the UV-blue window (λ_EX_ = 360 nm, λ_EM_ = 448 nm), and was reached in all cases after 15–20 min UVA irradiation in radioimmunoprecipitation assay (RIPA) lysis buffer (Figure 4A, top panels). This suggested that the intrinsic photoreactivity of the AZC tag, constituting a non-colligative property, could allow for a reproducible behavior in various media, such as cells and lysates, in a time-dependent but concentration-independent fashion. Moreover, control experiments using cpd. **6** as exemplary of AMC fluorescence showed that its emission intensity was BSA independent, whereby it increased in a dose–response manner with cpd. **6** concentration up to 50 µM (Figure 4A, bottom panels). Regarding electrophoretic analysis, the dose–response profile obtained in the denaturing conditions of the PAGE (samples were heated with 1% SDS and gels run in the presence of 0.1% SDS) precluded the detection of labile non-covalent complexes, establishing the irreversible nature of the fluorogenic reaction between BSA and AZC-GA **5** (Figure 4B).

### 2.4. Fluorogenic Photoactivation Labeling of Live P. falciparum-Infected Erythrocytes with AZC-GA 5 

**Figure 5:**
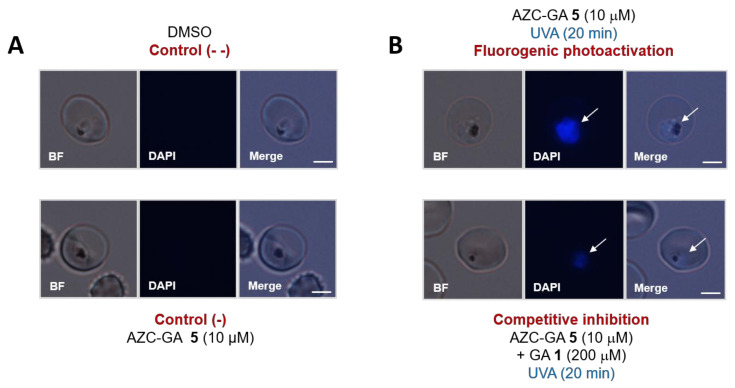
Photoalkylation of live 3D7 *P. falciparum* trophozoite blood-stages by AZC-GA **5** ± GA **1**. (**A**): Negative controls consisted of parasites treated with dimethyle sulfoxide (DMSO) or with non-photoactivated AZC-GA **5**. (**B**): Fluorogenic experiments consisted of parasites treated with photoactivated AZC-GA **5** in the absence or presence of excess GA **1**. Images were obtained on an Olympus BX60F-3 microscope using DAPI excitation channel (λ_EX_ = 359 nm). All UVA photoactivations were performed on ice (0–4 °C) for 20 min. Fluorescence was recorded as seen with the naked eye. Images were taken using identical exposure times (132 ms) and are representative of several independent experiments (*n* = 2–4, see Figure S1 in the Supplementary Materials). White arrows indicate the parasites. Scale bars represent 2 µm.

Following the expected behavior of AZC-GA **5** in model conditions, we turned to study this chemical probe in relevant *P. falciparum* systems. Using the parasite laboratory strain 3D7 and a low concentration of 10 µM AZC-GA **5** (corresponding to ca. two-fold its IC_50_ value), a strong fluorogenic labeling within live trophozoites upon photoactivation under the optimized conditions previously determined with BSA (i.e., 20 min UVA irradiation at 0–4 °C) could be detected using the excitation channel for 4′,6-diamidino-2-phenylindole (DAPI, λ_EX_ = 359 nm) (Figure 5B, top panel). In the same conditions, negative controls lacking AZC-GA **5** or performed without photoactivation showed a complete absence of fluorescence (Figure 5A). Importantly, a complete absence of photolabeling by AZC-GA **5** was observed in the cytosol of infected erythrocytes (Figure 5B, top panel) but also uninfected erythrocytes (data not shown), showing the complete specificity of the probe towards *P. falciparum* blood-stages and not their host cell. Furthermore, the photolabeling of live *P. falciparum* cells by **5** was competitively inhibited upon co-administration of a 20-fold excess GA **1**, with a strong reduction in intra-parasite fluorescence (Figure 5B, bottom panel). These results suggested that AZC-GA probe **5** was of excellent pharmacological specificity despite the introduction of a bulky AZC tag, and that it was targeting binding sites common to the natural PPAP within *P. falciparum*.

### 2.5. Fluorogenic Photoactivation Labeling of Fresh P. falciparum Lysate with AZC-GA 5 

**Figure 6:**
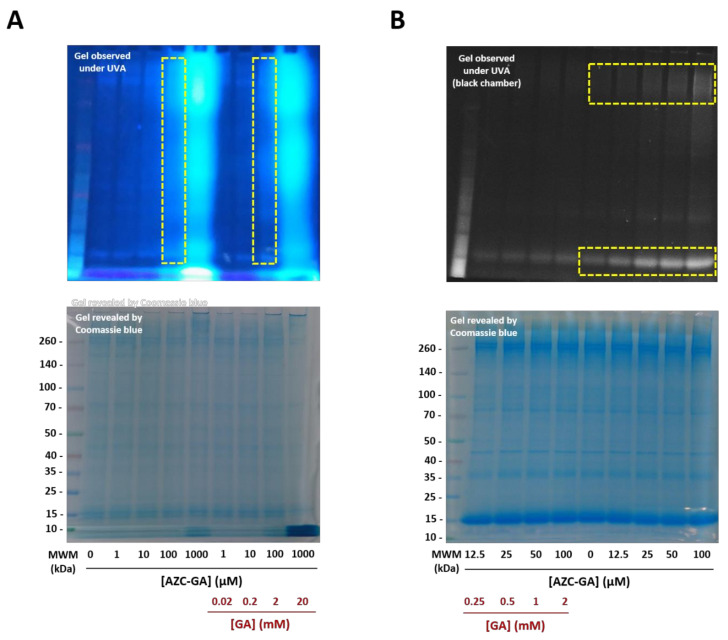
Photoalkylation of asynchronous 3D7 *P. falciparum* cell lysate by AZC-GA **5** ± GA **1**. (**A**): 0–1 mM range; lysate concentration was 1 mg/mL total protein. (**B**): 0–100 µM range; lysate concentration was 5 mg/mL total protein. Noteworthy, various plasmodial proteins show slight blue autofluorescence in the absence of AZC-GA **5** (lanes 0 µM). Fluorescence revelation was performed using the UV bed of an E-Box VX2 scanner (Vilber-Lourmat) and pictured as seen with the naked eye (**A**) or under limited exposure in a black chamber (**B**). All photoactivations were performed on ice (0–4 °C) for 20 min. MWM = molecular weight marker.

We then studied the photoalkylative properties of AZC-GA **5** against native plasmodial proteins within a fresh lysate of asynchronous 3D7 *P. falciparum* parasites. Using a range of 0–1000 µM **5** and our optimized conditions of irradiation, a strong fluorogenic labeling of parasite proteins upon UVA photoactivation could be obtained between 100 and 1000 µM **5**, with no or faint labeling at inferior concentrations. Intensive proteome labeling occurred, with fluorescence detected from low- to high-molecular-weight proteins (Figure 6A). The photolabeling at 100 µM AZC-GA **5** was competitively inhibited upon co-administration of a 20-fold excess GA **1** with a significant reduction in fluorescence, whereas labeling at 1000 µM **5** remained non-competed and displayed a saturation profile (Figure 6A). These results were refined using a narrower range of 0–100 µM AZC-GA **5**, yielding a dose–response profile of protein photolabeling upon observation under UV light in a black chamber. The labelings were clustered in the high-molecular-weight zone (ca. 260 kDa) and associated to a low-molecular-weight band (ca. 15 kDa), and were significantly abolished by excess GA **1**, displaying profiles that were similar to that of the control in absence of AZC-GA **5** (Figure 6B). 

## 3. Materials and Methods

### 3.1. Chemistry

#### 3.1.1. General Information

Reagents and anhydrous solvents were of high grade (Merck-Sigma, St. Louis, MO, USA) and used as received. 2-(7-Amino-4-methyl-2-oxo-2H-chromen-3-yl)acetic acid **6** and bovine serum albumin (BSA, fraction V, 66 kDa) were purchased from Merck-Sigma. RIPA buffer was prepared according to [32]. Column chromatography was performed using silica gel 60 (9385 Merck). Thin Layer Chromatography (TLC) was performed on aluminum plates coated with silica gel 60F_254_ (554 Merck), visualized with UV light (254 and 366 nm) and revealed with sulfuric vanillin and/or ninhydrin. NMR spectra (^1^H, ^13^C, COSY, HMBC, and HSQC) were recorded on an Advance Bruker 400 MHz spectrometer (Rheinstetten, Germany), using solvent signal as an internal standard (*d_6_*-DMSO: δ 2.50 and 39.5 ppm, *d_6_*-acetone: δ 2.05 and 29.8 ppm). High-resolution mass spectra (HRMS) were recorded in the ESI mode on an LCT Mass Spectrometer (Waters, Milford, MA, USA) equipped with a TOF analyzer. 

#### 3.1.2. Compound Synthesis

##### **Cpd. 7**: 2-(7-azido-4-methyl-2-oxo-2H-chromen-3-yl)acetic acid

To a well-stirred suspension of **6** (500 mg, 2.14 mmol) in water (8 mL) at 0 °C was added *p*-toluenesulfonic acid monohydrate (4.07 g, 21.4 mmol), yielding a clear yellow solution. The reaction mixture was protected from light, then a solution of sodium nitrite (590.6 mg, 8.56 mmol) and sodium azide (556.5 mg, 8.56 mg) dissolved in water (4 mL) was added dropwise. The mixture was stirred for 2 h at 0 °C then treated with 1N sodium hydroxide added dropwise until pH 7–8 was reached. The solution was extracted with AcOEt (8 × 10 mL), the organic phases were pooled, dried on sodium sulfate, filtered, and evaporated under reduced pressure. The crude product was retaken in MeOH (50 mL), silica (2.0 g) was added, and the mixture evaporated to dryness under reduced pressure. The adsorbed product was purified by column chromatography (CH_2_Cl_2_/MeOH 100:0 to 95:5 *v*/*v*), yielding **7** (195 mg, 31%) as a beige solid. R*_f_* (silica TLC) = 0.28 (CH_2_Cl_2_/MeOH 95:5 *v*/*v*); ^1^H-NMR (400 MHz, *d_6_*-DMSO) δ 7.79 (*d*, *J* = 5.2 Hz, 1H), 7.09 (*m*, 2H), 3.57 (*s*, 2H), 2.35 (*s*, 3H); ^13^C-NMR (100 MHz, *d_6_*-DMSO) δ 160.7, 152.8, 148.8, 142.9, 127.4, 119.0, 117.2, 115.9, 106.7, 100.2, 33.1, 15.2; ESI-HRMS [M + Na]^+^, *m*/*z* 282.0491 (calculated), *m*/*z* 282.0463 (found); [M − H]^−^, *m*/*z* 258.0515 (calculated), 258.0487 (found).

##### **Cpd. 8**: 2,5-Dioxopyrrolidin-1-yl 2-(7-azido-4-methyl-2-oxo-2H-chromen-3-yl)acetate

To a stirred suspension of **7** (166 mg, 0.64 mmol) in THF (25 mL) at 0 °C and protected from light, *N*-hydroxysuccinimide (75 mg, 0.65 mmol), 4-dimethylaminopyridine (4 mg, 32.7 µmol), and then *N*-(3-dimethylaminopropyl)-*N*′-ethylcarbodiimide hydrochloride (125 mg, 0.65 mg) were added. The reaction mixture was allowed to warm up to room temperature and stirred for 15 h. The obtained white suspension was filtered, the precipitate rinsed with ice-cold THF (3 × 2 mL), and the filtrates pooled and evaporated under reduced pressure. The crude product was purified by column chromatography (CH_2_Cl_2_/EtOAc 95:5 to 80:20 *v*/*v*), yielding **8** (118 mg, 52%) as a beige solid. R*_f_* (silica TLC) = 0.50 (CH_2_Cl_2_/EtOAc 85:15); ^1^H-NMR (400 MHz, *d_6_*-DMSO) δ 7.85 (*d*, *J* = 8.6 Hz, 1H), 7.14 (*s*, 1H), 7.12 (*d*, *J* = 8.5 Hz, 1H), 4.08 (*s*, 2H), 2.78 (*s*, 4H), 2.44 (*s*, 3H); ^13^C-NMR (100 MHz, *d_6_*-DMSO) δ 170.3, 166.5, 160.3, 153.1, 150.7, 143.6, 127.7, 116.9, 116.2, 116.1, 106.9, 29.9, 25.6, 15.4; ESI-HRMS [M + Na]^+^, *m*/*z* 379.0655 (calculated), 379.0640 (found).

##### **AZC-GA 5**: 2-Hydroxy-4-((6R,7S)-2-hydroxy-6-methyl-1,5,7-tris(3-methylbut-2-en-1-yl)-6-(4-methylpent-3-en-1-yl)-4,9-dioxobicyclo [3.3.1]non-2-ene-3-carbonyl)phenyl 2-(7-azido-4-methyl-2-oxo-2H-chromen-3-yl)acetate

To a stirred solution of GA **1** (20 mg, 33.2 µmol) in CH_2_Cl_2_ (1 mL) protected from light at room temperature, **8** (12 mg, 33.7 µmol) was added, and then NEt_3_ (5 µL, 35.6 µmol) and 4-dimethylaminopyridine (1 mg, 8,2 µmol). The reaction mixture was stirred for 15 h, and then evaporated under reduced pressure. The crude product was purified by column chromatography (CH_2_Cl_2_/cyclohexane/EtOAc 50:30:20 *v*/*v*/*v*), yielding **5** (16 mg, 57%) as a yellow resin. R*_f_* (silica TLC) = 0.46 (CH_2_Cl_2_/cyclohexane/EtOAc 50:30:20 *v*/*v*/*v*); ^1^H-NMR (400 MHz, *d_6_*-acetone) δ 7.91 (*d*, *J* = 8.6 Hz, 1H), 7.46 (*s*, 1H), 7.38 (*d*, *J* = 8.5 Hz, 1H), 7.14 (*d*, *J* = 8.6 Hz, 1H), 7.10 (*s*, 1H), 6.96 (*d*, *J* = 8.5 Hz, 1H), 5.26 (*t*, 1H), 5.11 (*t*, 1H), 5.01 (*br*, 1H), 4.94 (*t*, 1H), 2.82 (*br*, 2H), 2.71 (*d*, 2H), 2.54 (*br*, 2H), 2.14 (*s*, 1H), 1.92 (*m*, 3H), 1.71 (*s*, 3H), 1.67 (*s*, 9H), 1.64 (*s*, 3H), 1.60 (*s*, 3H), 1.47 (*s*, 3H), 1.29 (*s*, 3H); ^13^C-NMR (100 MHz, *d_6_*-acetone) δ 207.4, 194-195 (two missing), 193.8, 168.1, 160.6, 153.6, 153.5, 149.4, 143.5, 138.1, 133.8, 132.1, 131.5, 129.8, 128.5, 127.1, 124.5, 124.1, 119.9, 118.3, 117.4, 115.7, 115.6, 106.7, 54.1, 50.7, 40.4, 39.6, 35.6, 32.7, 32.3, 30.9, 29.7, 28.2, 26.0, 25.7, 25.3, 25.0, 24.9, 22.4, 17.4, 17.3, 17.1, 16.7, 14.8; ESI-HRMS [M + H]^+^, *m*/*z* 843.4095 (calculated), 843.4039 (found).

#### 3.1.3. Fluorimetric Study

BSA solutions (0 to 8 mg/mL) were prepared from an 8 mg/mL stock solution in RIPA buffer deprived of dithiothreitol, ATP, as well as phosphatase and protease inhibitors. Solutions of AZC-GA **5** and Cpd. **6** (0 to 100 µM) were prepared from a 100 µM stock solution in identical RIPA buffer. Components were mixed in the adequate proportions in 1-mL-capacity Eppendorff tubes and transferred to 1-mL-capacity quartz cuvettes of a 10-mm path length (Hellma Analytics, Müllheim, Germany). Fluorescence was recorded on a Xenius XML spectrofluorimeter (SAFAS) at 23 °C before and after photoactivation for various times at 0–4 °C, using a UVA 100 W lamp (Jena Analytiks, Jena, Germany) positioned right above the closed cuvettes lying on an ice bed, using λ_EX_ = 360 nm and λ_EM_ = 448 nm as the detection settings.

### 3.2. Biology

#### 3.2.1. General Information

4-(2-Hydroxyethyl)-1-piperazine-ethanesulfonic acid (HEPES) buffer and phosphate buffered saline (PBS), sodium *ortho*-vanadate, d-glucose, sodium bicarbonate, sodium chloride, sodium fluoride, hypoxanthine, l-glutamine, methanol, DAPI, dihydroartemisinin, and saponin were purchased from Merck-Sigma. RPMI 1640 medium and AB human serum were purchased from Gibco (Carlsbad, CA, USA). RIPA buffer was prepared according to [32].

#### 3.2.2. Parasite Culture

The *P. falciparum* 3D7 strain was maintained in human erythrocytes (O^+^, provided by Etablissement français du sang, EFS, France) at 5% hematocrit, suspended in complete culture medium (RPMI 1640 supplemented with 25 mM HEPES, 20 mM d-glucose, 25 mM sodium bicarbonate (Merck-Sigma), 0.4 mM hypoxanthine, 5 mM L-Glutamine, and 10% AB human serum), according to the procedures from “Methods in Malaria Research” (https://www.beiresources.org/portals/2/MR4/Methods_In_Malaria_Research-6th_edition.pdf), adapted from [33]. Parasite cultures were kept at 37 °C in a gaseous environment composed of 5% CO_2_, 5% O_2_, and 90% N_2_. The culture medium was changed daily, with control of parasitemia using an Axioskop light microscope (Zeiss, Thornwood, NY, USA) under oil immersion, after fixing thin blood smears by methanol and staining with Diff-Quik^TM^ stain Set (RAL Diagnostics, Martillac, France).

#### 3.2.3. IC_50_ Measurement

The 50% inhibitory concentrations (IC_50_) determination test was carried out by isotopic 42 h ^3^H-hypoxanthine incorporation assays as previously described [34] with minor modifications. Briefly, *P. falciparum* cultures at ring-stage were highly synchronized by two consecutive treatments with 5% sorbitol in PBS (*v*/*v*) at 40-h intervals and diluted down to 0.3–0.5% parasitemia and 2% hematocrit. Parasites were dispensed into 96-well plates containing 14 serially diluted concentrations of drug ranging from 0 to 50 µM for GA **1**, AZC-GA **5** and cpd. **7**, and from 0 to 952 nM for dihydroartemisinin, and incubated as described above in the presence of 5% ^3^H-hypoxanthine (Perkin Elmer, Hopkinton, MA, USA) for 42 h. ^3^H-hypoxanthine uptake was then evaluated by scintillation counting (Top Count NXT, Perkin Elmer) and results were expressed as the inhibitory concentrations IC_50_, defined as drug concentrations at which 50% of ^3^H-hypoxanthine incorporation was inhibited compared with drug-free controls. IC values were established by non-linear regression with ICEstimator software (http://www.antimalarial-icestimator.net/) [35,36]. All tests were done in triplicates.

#### 3.2.4. Photoactivation Fluorogenic Microscopy

To assess the localization of AZC-GA probe **5** in 3D7 *P. falciparum*, infected erythrocytes (ca. 5% parasitemia) containing mainly trophozoites were transferred from complete culture medium to PBS by three cycles of 15 s microcentrifugation/PBS wash and kept on ice. Aliquots of the parasite suspension (200 µL) were transferred to 600-µL-capacity Eppendorf tubes and incubated with 10 µM AZC-GA **5** (from a 10 mM stock solution in DMSO) +/− 200 µM GA **1** (from a 200 mM stock solutions in DMSO) for 20 min at 23 °C, and then photoactivated for 20 min at 0–4 °C using a UVA 100 W lamp (Jena Analytiks) positioned right above the opened Eppendorf tubes. Negative controls consisted of parasites treated with 0.1–0.2% DMSO or with AZC-GA **5** but unexposed to UVA. Following irradiation, cells were quickly washed with cold PBS as described above, mounted, and observed in the bright field and using the DAPI excitation channel of an BX60F-3 microscope (Olympus, Tokyo, Japan).

#### 3.2.5. Parasite Lysate Preparation

Asynchronous 3D7 *P. falciparum* parasites were cultivated in several 75-cm^2^ flasks (Life Science) at 5% hematocrit in a gassed incubation chamber as described previously. Medium was changed daily until parasitemia reached 7–10%. Cultures were transferred to 50 mL Falcon tubes and centrifuged at 500× *g* for 5 min. The pellets were washed once with 25 mL PBS before the saponin lysis as previously described, with minor modifications [37]. Briefly, the pellets were resuspended in 25 mL freshly made saponin lysis buffer (consisting in 0.03% saponin *w/v* in PBS kept at 4 °C) and incubated on ice for 20 min. Erythrocyte-free parasites were pelleted by centrifugation at 4000× *g* for 5 min to discard the supernatant and repeatedly washed with PBS until no red color could be detected with the naked eye. The pellets were resuspended in 500 µL of parasite lysis buffer (consisting in RIPA buffer complemented with 10 mM β-glycerophosphate, 10 mM NaF, 0.1 mM sodium *ortho*-vanadate, 1 mM phenylmethylsulfonyl fluoride, 10 mM benzamidine, and Complex^TM^ protease inhibitors (Roche, Boulogne-Billancourt, France), and two brief sonications on ice (50% amplification, 20 s *on*, 40 s *off*) were applied. The homogenate was centrifuged at 13,000× *g* for 30 min at 4 °C to obtain a clear lysate. Total protein concentration was determined by the Coomassie Plus^TM^ Protein Assay (ThermoFisher, Waltham, MA, USA). Lysate quality was verified by SDS-PAGE on 4–12% gradient gel (NuPAGE^TM^ 4–12% Bis-Tris Gel, Life Technologies, Carlsbad, CA, USA).

#### 3.2.6. Covalent Protein Photolabeling in Fresh *P. falciparum* Lysate

Aliquots of freshly prepared cell lysate from asynchronous 3D7 *P. falciparum* parasites (100 µL, 1–5 mg/mL total protein) were transferred to 200-µL-capacity Eppendorf tubes. AZC-GA probe **5** (from a 10 mM stock solution in DMSO) +/− 20X GA **1** (from 200 mM or 2 M stock solution in DMSO) was added at various concentrations and incubated at 37 °C for 20 min in the dark, and then photoactivated for 20 min at 0–4 °C using a UVA 100 W lamp (Analytik Jena) positioned right above the opened Eppendorf tubes. Negative controls consisted of parasites treated with the corresponding quantities of DMSO (0.1–1.1%). All experiments were stopped by adding Laemmli buffer 5X (1X final concentration) and heated at 95 °C for 10 min before cooling down to room temperature. Covalently labeled proteins were separated by SDS-PAGE on 4–12% gradient gel as previously described, and fluorescence visualization was carried out with an E-Box VX2 scanner (Vilber-Lourmat).

## 4. Conclusions

Based on previous SARs in the GA **1** series, we rationally designed and synthesized a GA-derived photoactivatable fluorogenic probe, AZC-GA **5**, *en route* to studying the mechanisms of action of this natural PPAP in malaria parasites. Probe **5** showed an inhibitory activity against *P. falciparum* blood-stages in vitro that was superimposable to that of native GA **1**, validating its relevance as a molecular probe in the field. Furthermore, probe **5** exhibited a good level of pharmacological specificity in imaging and proteomic settings despite the introduction of a bulky AZC tag, suggesting that it was targeting binding sites common to the natural product GA **1** within *P. falciparum*. However, the protein fluorogenic labeling by AZC-GA **5** required rather high concentrations of probe **5** (25–100 µM, Figure 4 and Figure 6) and remained incompletely inhibited even in the presence of a large excess of competing GA **1** (Figure 5 and Figure 6), indicating a stable level of non-specific binding. Our results suggest that fluorogenic tags smaller and more photoreactive than AZC (e.g., 2-azidobenzoate [38]) should be introduced onto GA **1** to enable highly specific proteomic analysis of its targets in *P. falciparum*, by means of second-generation probes. To the best of our knowledge, our study represents the second example of a fluorescently-labeled PPAP devoted to biological interrogations, following the work on the localization of the anticancer agent gambogic acid in HeLa cells [25].

## Supplementary Materials

The following is available online, Figure S1: representative photoactivation images of live 3D7 *P. falciparum* trophozoite blood-stages by AZC-GA.
