# Supplementary material for: A Photoalkylative Fluorogenic Probe of Guttiferone A for Live Cell Imaging and Proteome Labeling in Plasmodium falciparum
Source: Molecules. 2020 Nov 4;25(21):5139. doi: 10.3390/molecules25215139 (PMC7663766; doi:10.3390/molecules25215139)
Supplement: Supplementary file 1 [file molecules-25-05139-s001.pdf]

A Photoalkylative Fluorogenic Probe of Guttiferone A for Live Cell Imaging and Proteome Labeling in *Plasmodium falciparum*

Romain Duval, Kevin Cottet, Magali Blaud, Anaïs Merckx, Sandrine Houzé, Philippe Grellier, Marie-Christine Lallemand and Sylvie Michel

ELECTRONIC SUPPLEMENTARY INFORMATIONS

Page 1: Summary

Page 2: Figure S1 (Representative photoactivation images of live 3D7 *P. falciparum* trophozoite blood-stages by AZC-GA **5** ± GA **1)**

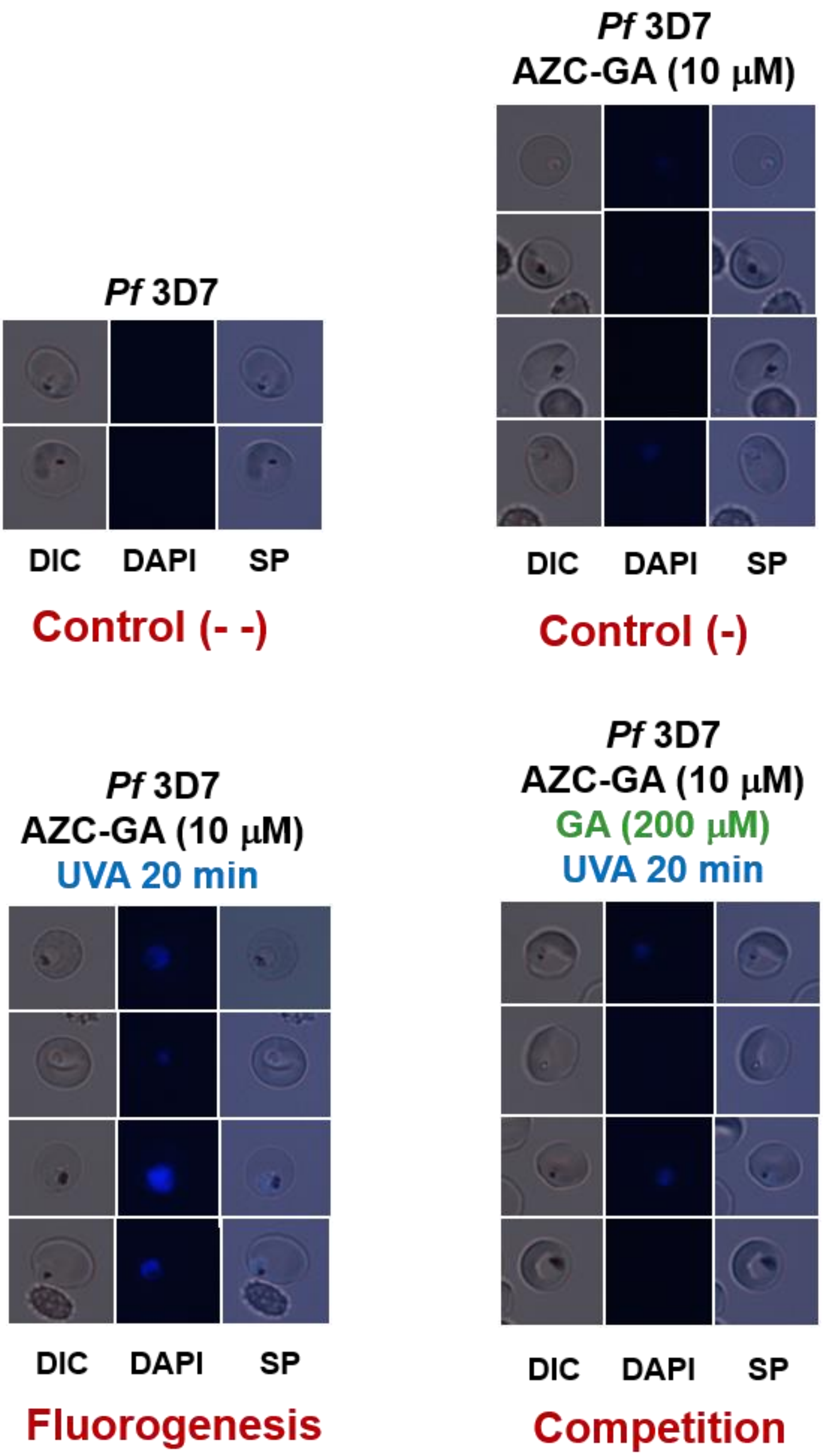

Figure S1: Representative photoactivation images of live 3D7 *P. falciparum* trophozoite blood-stages by AZC-GA **5** ± GA **1** (see Fig. 5 in the Article for experimental details).
